# Caregivers’ understanding and response to healthcare-associated infections in hospitalised children in Vietnam: a qualitative study

**DOI:** 10.1136/bmjpo-2025-004199

**Published:** 2026-02-12

**Authors:** Rebecca Lindblom, Ngoc Anh Tran, Van Anh Thi Dinh, Duyen Thi Kim Truong, Hằng Thị Thu Nguyễn, Tùng Anh Trần, Ngai Kien Le, Nhung Nguyen Thi Trang, I-CRECT-study group, Håkan Hanberger, Tung Viet Cao, Dien Minh Tran, Mattias Larsson, Linus Olson, Bent Jörgensen

**Affiliations:** 1 School of Global Studies, University of Gothenburg, Gothenburg, Sweden; 2 Sahlgrenska Academy, University of Gothenburg, Gothenburg, Sweden; 3 The Training and Research Institute of Child Health, Hanoi, Viet Nam; 4 Department of Infection Prevention and Control, Vietnam National Children’s Hospital, Hanoi, Viet Nam; 5 Cancer Center, Vietnam National Children’s Hospital, Hanoi, Viet Nam; 6 Neonatal Center, Vietnam National Children’s Hospital, Hanoi, Viet Nam; 7 Training and Research Academic Collaboration (TRAC) – Sweden – Vietnam, Hanoi, Viet Nam; 8 Director Board, Vietnam National Children’s Hospital, Hanoi, Viet Nam; 9 Hanoi University of Public Health, Hanoi, Viet Nam; 10 Department of Global Public Health, Karolinska Institute, Stockholm, Sweden; 11 Department of Women's and Children’s Health, Karolinska Institutet, Stockholm, Sweden

**Keywords:** Caregivers, Intensive Care Units, Neonatal, Developing Countries

## Abstract

**Background:**

The presence of family members caring for hospitalised children provides essential support and comfort, but it also poses a significant risk for the transmission of infectious pathogens. Vietnam is facing a growing issue with healthcare-associated infections (HAIs) and increasing antimicrobial resistance, reflecting a global trend. Children, particularly newborns with underdeveloped immune systems, are at increased risk.

**Aim:**

This study aims to explore the awareness of HAIs as well as motivational drivers towards infection prevention through the lens of the Health Belief Model (HBM) among family caregivers of patients at a children’s hospital in Hanoi, Vietnam.

**Method:**

Individual interviews, focus group discussions and observations were conducted with family caregivers (*n*=24) of children and newborns at the Oncology and Neonatal ward at Vietnam’s National Children’s Hospital from 15 January 2024 to 3 March 2024. Participants were chosen by purposeful sampling. Data were analysed by abductive, qualitative content analysis based on the HBM.

**Result:**

Awareness of HAIs varied, with some misconceptions about transmission. Recognising HAIs as serious health threats beyond basic hygiene knowledge boosted motivation to follow infection prevention and control (IPC) measures as well as longer hospital stays and social influences. Increased awareness of infection risks was linked to stronger adherence, aligning with HBM principles.

**Conclusion:**

Family caregivers play a vital role in infection prevention, yet their awareness of HAIs is inconsistent and sometimes limited by misconceptions. This study highlights that perceiving HAIs as serious health threats is a key motivation of improved hygiene compliance, as in line with the HBM. Family caregivers play a vital role in maintaining a safe hospital environment, and their involvement in IPC strategies should be actively researched, supported and strengthened.

**WHAT IS ALREADY KNOWN ON THIS TOPIC**

- The role of family caregivers is crucial in paediatric care, but their role as a pathway for the transmission of pathogens is not yet well documented.

**WHAT THIS STUDY ADDS**

- This qualitative study shows that the infection prevention and control role of family caregivers in paediatric hospital settings is generally neglected.
- By applying the health belief model, the study shows that family caregivers constitute a potential contribution to healthcare-associated infections.

**HOW THIS STUDY MIGHT AFFECT RESEARCH, PRACTICE OR POLICY**

- Family caregivers should be recognised as key players in policy and research aiming at reducing healthcare-associated infection.

## Introduction

 Antimicrobial resistance (AMR) is one of our time’s greatest threats to child survival and to global health since antibiotics have saved countless millions of lives in recent decades. In 2019, 254 000 of the 1,27 million cases of child mortality all over the world were directly linked to AMR, with approximately 99% of the children being from low- and middle-income countries (UN, 2023).^1^ This threat is especially severe among vulnerable populations, including neonates and children with underdeveloped immune systems.^2^ This made the study team address the possible causes of transmissions and where infection prevention and control (IPC) needed to be strengthened.

In Vietnam, AMR is increasing and poses a heavy burden on society in terms of morbidity, mortality and economic impact, with hospitals representing significant locations of infection spread over length of stay.^2 3^ Carbapenem-resistant Enterobacteriaceae (CRE) are among the most concerning groups of drug-resistant pathogens, highlighting the severity of this challenge. In 2019, CRE colonisation was reported in nearly half of Vietnam’s patients in hospital, with prevalence increasing during hospitalisation from 13% on the first day to 89% by day 15.^2^ There is an urgent need for life-saving interventions to limit the spread of resistant microbes and to strengthen IPC strategies, including recognition of the important role family caregivers play in paediatric care and their influence on preventing AMR transmission within hospital settings.^3^

When a child or newborn is hospitalised, family involvement in the child’s care has been shown to be crucial for the patient’s recovery as well as the family caregivers’ well-being.^4^,^6^ Even though family caregivers play a significant role as informal caregivers in the hospital, their role in IPC and transmission of healthcare-associated infections (HAI) is not as widely studied as formal healthcare staff, which creates a knowledge gap that needs to be addressed.^5^

To enhance our understanding of family caregivers’ motivation to contribute to IPC measures and awareness of HAI, this study is grounded in the perspectives of the family caregivers themselves through the lens of the Health Belief Model (HBM). The HBM emphasises that awareness of a health threat shapes behaviour through perceived risk and the seriousness of its consequences. Therefore, if individuals believe they are at risk and the threat is severe, they are more likely to take preventive action. Through a linkage of key concepts, the model can be used to investigate why individuals take action, or not, to manage to prevent, screen for or control illness conditions. The key concepts presented in the HBM are perceived susceptibility, perceived severity, perceived benefits, perceived barriers, cues to action, self-efficacy and modifying variables.^7^,^9^

The qualitative method was chosen as it is holistic and in-depth, exploring people’s values and lived experiences and how these values are expressed.^10^ Through a qualitative approach, this study aims to explore the motivation towards IPC practice as well as awareness of HAI, through the lens of the HBM, among family caregivers of patients at a children’s hospital in Hanoi, Vietnam.

## Methodology

### Study design

The research was a non-experimental, qualitative study with an abductive approach based on the HBM. Data were gathered through observations, focus group discussions and individual interviews. A semi-structured interview guide was used during the focus group discussions and individual interviews covering aspects of caregivers’ perception of HAI and IPC based on the theory of the HBM. The data were analysed using abductive qualitative content analysis. The results are presented thematically using the HBM rather than being organised by data collection methods.

### Setting

This study took place at Vietnam National Children’s Hospital in Hanoi, which is one of the leading paediatric hospitals in Vietnam with approximately 6000–7000 inpatients and outpatients per day. Data were collected at one of the hospitals' oncology wards and one of the neonatal wards from 15 January to 3 March 2024. Each of these wards accommodates ~50 patients each, the majority of whom stay in shared rooms of about five to six patients.

The interviews were conducted by two interviewers who worked as a team, consisting of the first author of this article and a colleague of the research project, where one had Vietnamese as a native language. The team was given direct feedback during data collection and hence could ask probing questions. The study team strictly followed the Vietnam National Children's Hospital (VNCH’s) hygiene routines to mitigate contributing to HAI. Also, see table 1 for interview methodology.

**Table 1:** Summarises the characteristics of study participants and the data collection methods

Category	Details
Total no. interviewees	24 individuals
Ward distribution	Oncology ward: 14 participants Neonatal ward: 10 participants
Relationship to patient	Mothers: 18 Grandmothers: 5 Aunt: 1
Participant age range	23–67 years
Patient age range	5 days to 13 years
Focus group discussion	5 in total
Participants per focus group	2–3 participants
Focus group duration	15–30 min
Focus group ward distribution	Oncology ward: 4 Neonatal ward: 7
Individual interviews	11 in total
Individual interview duration	9–20 min
Individual interview ward distribution	Oncology ward: 4 Neonatal ward: 7

### Participants and informants

Participants were selected through purposeful sampling as these patient groups tend to have longer hospital stays and furthermore are particularly sensitive to HAI related to suppressed and/or underdeveloped immune systems. Participants sharing patient rooms in the wards formed the focus groups. For further info about the characteristics of study participants and the data collection methods, see (table 1).

The inclusion criteria for the participants were: being a family caregiver to a child or a neonate admitted to the inpatient Neonatal or the Oncology ward, having a hospital stay of more than 3 days, being over the age of 18 and having the ability to speak fluent Vietnamese. It was difficult to determine the participants’ total length of stay, as some patients had previous admissions before the current hospitalisation. Exclusion criteria included patients admitted to intensive care; those in isolation for infectious diseases (eg, rhinovirus, respiratory syncytial virus or other airborne diseases); and patients whose caregivers were temporary replacements present for only 1–2 hours rather than the primary hospital caregiver. Regarding the number of focus group discussions and interviews, data collection continued until saturation was achieved, that is, when answers among participants recurred and no significantly new information emerged.

It was noted by the informants that the data would possibly be skewed due to the knowledge gap between family members in the Neonatal and the Oncology ward, since family caregivers at the Oncology ward have longer hospital stays. However, it was chosen to include participants from both wards to gain a broader representation of family caregivers. It was also an active choice to include family caregivers to patients of a wide range of ages, since it was observed that the patients were significantly dependent on the family caregiver regarding age.

Focused ethnography was used for the observations, as this approach proved particularly suitable for the ward setting. In contrast to macro ethnography, it emphasises observing a smaller, limited group, or situations in a specific context, for example, a hospital ward. Focused ethnography enables observations of the field for a shorter period. The study highlights the emic and etic perspectives, since the data provide the participant’s perception of the phenomena as well as the researcher’s observations and interpretations.^11^

### Patient and public involvement

To get access to the wards, the research project was presented to the staff members at the Neonatal and Oncology ward. Healthcare staff asked questions and provided feedback about the project and approved the presence of the study team’s attendance in the wards and prepared the participants for the study team’s appearance at the wards. The interviews were conducted in the patients’ rooms within the wards to ensure that family members did not have to move or interrupt the care of their babies or children, allowing the interviews to be conducted in their natural environment.^12^

As informants, the healthcare staff from the IPC department and the Oncology- and Neonatal ward provided valuable insights about the context, sharing their knowledge and challenges regarding hygiene routines among family caregivers. Their contribution formed the foundation of the interview guide, and with their insights and triangulations of information, they enhanced the validity of the study.

### Analytical approach to the data

Field notes were taken with a pen and notebook, and interviews and focus group discussions were audio-recorded and then transcribed by using the AI-generated software Transkriptor, a service that provides audio file transcriptions, with the company based in Dubai. Thereafter, translated transcriptions were reviewed by a colleague with Vietnamese as native language.

Data were analysed by abductive qualitative content analysis according to Hsieh and Shannon^13^ using the HBM as framework. The analytical process of abductive qualitative content analysis began by identifying the key concepts of the HBM as initial coding categories. The process followed by reading the raw material multiple times to become familiar with the collected data. Next, the data that represented the key concepts were highlighted and later categorised by using the predetermined codes.

### Methodological considerations

The HBM has been useful in predicting hygiene behaviour among individuals since it explains factors that can affect the probability of adapting preventive behaviours. It has been an effective theoretical framework for designing educational interventions in healthcare settings to increase compliance with hygiene practices. According to the HBM, strengthening individual apprehension will increase the motivation to adopt and maintain preventive hygiene behaviours.^12^ The influences of behaviour change are complex and multifaceted, but crucial in designing programmes to improve hygiene compliance.^14^ When evaluating interventions and policies, stakeholders at all levels should be involved; hence the choice of interviewing family caregivers instead of healthcare staff.^15^

Potential bias from the interviewer effect may have influenced participants’ responses and behaviours during observations.^16^ The study leaders were two Swedish and Vietnamese women in their 20s with backgrounds in nursing, public and global health. To mitigate interviewer-related bias, measures emphasising respect were used, including active listening and maintaining a calm presence. Conducting research in a different cultural context requires cultural relativism, avoiding comparison, judgement or valuation of cultures.^17^ Accordingly, limited demographic data (eg, educational level) were collected, as such questions could compromise interviewer–participant trust.

SRQR reporting guidelines^18^ were used to draft this manuscript, and the SRQR reporting checklist (2) when editing, included in online supplemental appendix.

## Results

### Perceived benefits, perceived barriers and self-efficacy

Regarding the identified perceived benefits, there was a verbal consensus among participants that hygiene compliance was recognised as a beneficial measure to protect patients from HAI. Some participants expressed that they believed consequences for not maintaining proper hygiene could be infection, which could lead the child into a worse condition and hence postpone planned treatment and cause delay of recovery. One family caregiver expressed the fear that low compliance towards hygiene routines could lead to mortality.

Participants expressed high self-efficacy and perception of knowledge towards hygiene routines, and none of the interviewed participants mentioned any difficulties or provided suggestions for improvement. Family caregivers received hygiene education at the wards and family members were invited to attend an educational introduction held by medical doctors and registered nurses working at the IPC department.

The hospital area was decorated with hand hygiene promotion, as well as posters regarding hygiene practices such as in common areas outside, in elevators, at the wards and over sinks. The posters were mentioned by family caregivers as cues of acting towards hygiene routines. At the Oncology and Neonatal ward, there were hand sanitisers placed at the entrance of every room which made them available to use before and after visiting patient’s rooms.

Thus, there are not many obstacles or barriers in keeping things hygienic. /…/ There are illustrations on the walls of the hospital room for hand hygiene instructions. I also wash my hands very frequently, so I remember how to do it correctly. I don’t feel like there is anything more I need to know.- Family Caregiver Individual Interview number 5 at the Neonatal ward

### Perceived susceptibility and severity

Even though there was a spoken consensus among participants that hygiene practice and regulations were easy to perform and considered as a life-saving measure, participants expressed varying degrees of perceived susceptibility and severity regarding the transmission of infections in the hospital environment. Several participants from the Oncology ward articulated concerns about the potential risk of infections at the hospital and mentioned external risk factors such as crowded hospital areas, playrooms and shared rooms. Participants expressed thoughts about the premature babies and the children being young and vulnerable to infections, and family caregivers from the Oncology ward explained that the children are at various risks of infections depending on physical condition such as before and after treatment with chemotherapy that causes immunosuppression.

There will be a risk of cross-infection because it cannot be avoided. Because most of the children here are treated with chemicals, when chemicals enter their bodies, the children will be immunocompromised. Even though the nurses here are very hygienic, the children’s immune system is actually too weak. So sometimes even a very minor source of disease can still infect them and spread to other children. There is still some risk, but the healthcare staff have also limited the risk as much as possible. /…/Only when my child is healthy can he go out to the room to play. - Family Caregiver Individual Interview number 10 at the Oncology ward

Some family caregivers expressed feeling safer at the hospital compared with being at home and the word “secure” was mentioned several times during the interviews. One participant expressed feeling that the hospital was 100% hygienic and several participants stated that the hospital was cleaner than their homes. Furthermore, some did not report any concerns as long as they were staying at the ward or in the patient’s room. One family caregiver reflected on the isolation of patients within the hospital ward, explaining that this seclusion minimises the introduction of new viruses and bacteria and therefore no new infections can occur at the hospital. According to this participant, the primary source of new infectious pathogens stems from incoming patients and their families.

In the wards, each patient shares a bed with one adult and in some cases, patients share beds, which creates a situation where two patients and two adults share one hospital bed of about 90 cm × 215 cm. Sharing of beds was not unusual at the neonatal ward. One of the wards had a shared kitchen which was without a sink, so family caregivers used the shared bathrooms to wash kitchen equipment. The ward kitchen also did not have a dining area, and meals were observed to be taken in patients’ rooms.

Regarding the sharing of beds and rooms, family caregivers highlighted their confidence in healthcare staff’s efforts in assigning beds based on disease groups. For example, patients with meningitis share rooms with other patients that suffer from meningitis. The perception among some family caregivers regarding this strategy is that it helps to mitigate the risk of infections and that the disease is not easily transmitted as long as it is not transmitted by air.

With my efforts in maintaining hygiene standards and with the healthcare staff assigning beds according to the disease group, with babies having the same disease staying in the same room, I am not concerned about the risks of infections. - Family Caregiver Individual Interview number 4 at the Neonatal ward.

Two babies were assigned to the same bed. /…/I trust the doctor’s decision. If there is any risk of infection, or the baby has any problems, the doctor will arrange for the baby to move to another room.- Family Caregiver Individual Interview number 5 at the Neonatal ward

During time spent observing and interacting in conversations with family caregivers, there was a frequent usage of mobile phones among family members at the wards, as well as among the children. Phones were often found lying next to the neonates in the hospital beds at the Neonatal ward, and some had phone cases made of various materials. It was observed that phones were touched after diaper changes without washing or sanitising hands afterwards and then proceeded to touch facial masks and other surroundings.

A nurse at the neonatal ward explained that the main challenge regarding hygiene compliance among family caregivers is to make sure that they understand the importance of hygiene routines. The nurse experienced that the higher risk awareness family caregivers expressed, the better they were at performing hygiene according to routine. This goes in line with the concept of the HBM as well as previous studies, where it is shown that improvement in perceived susceptibility and perceived severity will increase people’s motivation to adopt, as well as maintaining, preventive health behaviours such as hand hygiene.

### Impact of the social environment

Throughout the hospital stay, participants described an adaptive process where they acquired new hygiene habits and skills, gradually becoming accustomed to the hospital environment and developing routines aimed at minimising infection risks. They emphasise the hands-on nature of their learning experience, where they actively engage in hygiene practices and learn by doing.

So, if there is any problem, the mothers will directly give feedback to each other, because we are in the same room together. If, for example, there is a mother whose hygiene awareness is not good, she will also be given feedback. We also have to understand that it is partly for our children, but it is also for other children. -Family Caregiver Individual Interview number 10 at the Oncology ward

Family caregivers also mentioned communication channels such as Zalo group chats for the exchange of tips, experiences and concerns among caregivers. The social interaction further strengthens their sense of collective responsibility for maintaining hygiene standards and protecting their children from infections.

## Discussion

Overall, the findings align with previous research and, when interpreted through the HBM, indicate that family caregivers demonstrate low perceived susceptibility and perceived severity regarding HAI. Limited knowledge and awareness of HAI and their consequences were common, alongside misconceptions about infection transmission. Notably, none of the participants mentioned AMR, and only one identified HAI as a potentially fatal threat. Within the HBM framework, this suggests that HAI and AMR are not perceived as prominent health threats, which may reduce motivation to engage in preventive behaviours. One explanation for the low perceived threat may be that information is being provided under stressful circumstances or without reinforcement. Additionally, the asymptomatic nature of AMR may further reduce perceived susceptibility, as participants appeared to prioritise risks associated with visible or symptomatic infections, such as airborne diseases. These findings highlight the importance of repeated and contextually appropriate information to strengthen risk perception and understanding.

The awareness of HAI is shaped by various factors, including social influences, educational exposure and cues to action. As participants spend more time in the hospital, they gradually become more receptive to these influences. The difference in length of hospital stays between the Neonatal and the Oncology wards illustrates this well. Caregivers at the Oncology ward, who had a longer hospital stay, tended to demonstrate and express a higher awareness of the risks associated with HAI. Even though isolation of infected patients has advantages from an IPC perspective, sharing rooms with experienced family caregivers and engaging with healthcare staff should not be underestimated. It offers valuable opportunities for accumulating knowledge and awareness about HAI, as well as motivation, from the social environment. Social interactions also appear to reinforce a collective sense of responsibility for maintaining hygiene standards and protecting children from infections.

Furthermore, another social instrument that was frequently used was the participants’ mobile phones. Smartphones and tablets are an important source of entertainment for patients who do not have the energy to leave their hospital beds, as well as for family caregivers to keep in touch with others while spending time at the hospital. The results show us that family caregivers use smartphones as a tool to gain new knowledge about hygiene practices. Using smartphones for educational interventions could be used in policy making and further explored in future studies. Although smartphones did not seem to be recognised as a tool of infection spread among participants. There are measures that can be provided by hospitals to mitigate the trade-off regarding IPC practice and psychosocial factors such as implementing surface disinfection in every ward and to exclude phone cases from materials that cannot maintain a high standard of hygiene.

The complexity noted by the collected data is that low self-efficacy, from an HBM perspective meaning the expressed fear of not being in control of potential cross-infections, can motivate individuals to take a more active leading role in hygiene compliance. On the other hand, individuals who expressed high knowledge of the routines and guidelines regarding hygiene practices felt safe and experienced a high trust in healthcare staff, tended to take a more passive role in hygiene practice and stagnation in the development of new hygiene skills, relying solely on healthcare staff to protect their children. Further actions would be to strengthen the family caregiver’s role with a Family-Centred-Care approach, and to reduce the belief that healthcare staff can protect the child from infections better than the family caregiver itself.

Observations identified multiple barriers to proper hygiene compliance, including shared rooms, beds and bathrooms; washing kitchen equipment in bathroom sinks; and the absence of designated stations for sanitising personal belongings such as mobile phones. These conditions raise concerns about the feasibility of family caregivers adhering to hygiene routines. Addressing these external challenges by providing support and resources to maintain clean environments and personal items, including mobile phones and tablets, may help caregivers comply with hygiene practices. Although family caregivers are expected to participate in patient care and follow hospital hygiene routines, they remain a secluded group that should be explicitly considered in hygiene policies to reduce the spread of HAI.

The results show that information about hygiene routines such as posters above sinks does not compensate for the awareness of HAI. The result shows that participants who expressed awareness of HAI adopted extra precautions when the patient was in conditions of being extra vulnerable to HAI, such as during chemotherapy. This underscores the importance of targeting threat perception alongside behavioural instructions. Educational interventions grounded in the HBM should therefore emphasise not only how to perform hygiene practices, but why these actions are critical in preventing HAI and AMR. Future research should evaluate HBM-based interventions aimed at enhancing awareness, motivation and sustained preventive behaviours among family caregivers.

### Limitations and strengths

The inclusion criteria of being able to speak fluent Vietnamese lead to the potential exclusion of ethnic minorities who do not speak Vietnamese as their mother tongue.^19^

Although the study applies the HBM to reveal a broad spectrum of factors influencing IPC, its scope is still limited to a few wards and a limited time period. In terms of reliability and transferability, cultural factors need to be taken into account, and replicability can always be questioned. On the other hand, Family Centred Care and Kangaroo Mother Care are recognised methods having IPC consequences worldwide, which makes the study relevant in a larger global context.

This study is among the few that apply qualitative research within IPC practices through the lens of the HBM, highlighting the perspectives of family caregivers.

By answering the research questions, this paper has generated knowledge and shed light on family caregivers as key players in improving IPC in paediatric care and hence reducing the spread of AMR. Research that strives to decrease AMR and the need for antibiotics is crucial to act towards one of today’s most urgent global health issues and one of the greatest threats to children’s health and survival.

## Conclusion

This study explored family caregivers’ awareness of HAI and their motivation to engage in IPC practices through the lens of the HBM. Findings show that awareness of HAI varied among participants, with some misconceptions about the transmission of infection. Longer hospital stays, social interaction and exposure to hospital routines were associated with increased awareness and a stronger motivation to precautionary behaviour. Knowing why hygiene practice is important and not only how it is performed is an important incentive for taking action towards hygiene compliance and the will to gain new knowledge. As key figures in the care of hospitalised children, family caregivers play a vital role in maintaining a safe hospital environment, and their involvement in IPC strategies should be actively researched, supported and strengthened.

## Supplementary material

10.1136/bmjpo-2025-004199 online supplemental file 1

## Data Availability

Data are available upon reasonable request.
