# Supplementary material for: Caregivers’ understanding and response to healthcare-associated infections in hospitalised children in Vietnam: a qualitative study
Source: BMJ Paediatr Open. 2026 Feb 12;10(1):e004199. doi: 10.1136/bmjpo-2025-004199 (PMC12911701; doi:10.1136/bmjpo-2025-004199)
Supplement: Supplementary file 2 [file bmjpo-2025-004199.reviewer_comments.pdf]

PEER REVIEW HISTORY

BMJ Paediatrics Open publishes all reviews undertaken for accepted manuscripts. Reviewers are asked to complete a checklist review form and are provided with free text boxes to elaborate on their assessment. These free text comments are reproduced below.

ARTICLE DETAILS

**TITLE (PROVISIONAL)**	Caregivers' understanding and response to healthcare-associated infections in hospitalised children in Vietnam: A qualitative study
**AUTHORS**	Lindblom, Rebecca<br>Tran, Ngoc<br>Dinh, Van Anh<br>Truong , Duyen<br>Nguyễn, Hằng<br>Trần, Tùng<br>Le, Ngai<br>Trang, Nhung<br>study group, I-CRECT study group<br>Cao, Tung<br>Tran, Dien<br>Larsson, M.<br>Olson, Linus<br>Jörgensen, Bent

VERSION 1 – REVIEW

**REVIEWER NAME**	Krishnan, Vijay
**REVIEWER AFFILIATION**	Department of Health Research
**REVIEWER CONFLICT OF INTEREST**	No Conflict of Interest
**AI DISCLOSURE**	No, I have not used AI
**DATE REVIEW RETURNED**	07-Nov-2025

**GENERAL COMMENTS**	As a frequently ignored part of healthcare provision especially in more community-focussed societies, the participation of caregivers in healthcare is a topic that deserves attention. This is all the more important when the setting of the study involves children, and the risks are as severe as hospital-acquired infection in a population that is at significant risk.<br><br>It is also reasonable to address this question through a qualitative approach (to elicit responses and observations that might not be found in a more structured or quantitative study) an to use multiple kinds of information-gathering (focussed group discussions, interviews, observation).<br><br>To be useful to practitioners and researchers trying to develop a better understanding of the ways in which family caregivers may influence infection prevention and control, it would be needed to have new insights that are developed from the information gathered, and that can be generalised at least to other, similar settings. The authors should try not to focus too much on the specifics of the setting, and should avoid extensive summarisation of observations, so that they might identify common themes or ideas.

Specific comments on sections of the article, follow:

Introduction

1. relevance of paragraph 2 of the introduction? (lines 40 - 47). May be relevant to the overall project, but does not link to the current paper.

2. “also poses a severe risk for the transmission of infectious pathogen” ([“bmjpo-2025-004199_Proof_hi”, p. 6](zotero://select/library/items/EQ5TQGCL)) ([pdf](zotero://open-pdf/library/items/GP5VYQ3K?page=6&annotation=4ZX69BYK)) \- would be useful to quantify this risk in terms of standard statistics (eg. attributable fractions)

Methods

1. more information is needed on how the interviews were conducted, and what measures were taken to improve validity. Although some procedural factors (language, setting, stance) are mentioned, the authors report (FGDS: n= 5; 2-3 participants, 15-30 minutes; Interviews: n = 11; 9 - 20 minutes; lines 32 to 40, page 7) that the interactions were quite brief. It would be good to elaborate on specific steps that were used to elicit qualitative information and to confirm saturation (as this was the reported endpoint to terminate sample collection).

2. It is unclear how the three information sources (FGDs, IIs, Obsevartions) interacted each other. It is also unclear whether the interview material was used in any way to identify specific areas for elaboration.

3. it appears that there were at least two interview teams. how was saturation assessed here? what

4. Some material appears to be elicited from staff. The corresponding section in the methods (page 8; 10-16) does not provide much detail. for any interaction with staff. If staff were considered to be participants, please clarify this (how many interviews, were they also considered or FGDs or IIs? if not, what was the difference?

5. Please simplify and clarify the description of interviews in lines 37-44, page 7. the total number and distribution of interviews, along with participant characteristics, should be clearly described. at present, interview characteristics and participant cahracteristics are presented side-by-side.

6. what observations were made? very little anthropological observations/material are provided in the results or discussion. Mostly the observations presented are structural and institution-specific (layout, staffing, presence/absence of IPC material)

7. If a caregiver was excluded due to their not having sufficient contact, were any steps taken

8. Essential details of the sample (proportion from neonatal/oncology setting, length of stay on the date of the interview) are not available.

	## Results<br><br>1. please separate FGD results, Interview results, observations here. As a qualitative study, it is important to differentiate sources and acknowledging subjectivity. This is missing in the presentation of results.

**REVIEWER NAME**	Bouchoucha, Stephane L.
**REVIEWER AFFILIATION**	
**REVIEWER CONFLICT OF INTEREST**	No Conflict of Interest
**AI DISCLOSURE**	No, I have not used AI
**DATE REVIEW RETURNED**	09-Nov-2025

**GENERAL COMMENTS**	Thank you for the opportunity to review this manuscript. A very important topic, under researched.<br>The submission is well constructed and written. My only criticism would be that there are no obvious links made with the HBM in your discussion IT would be interesting to use the HBM as a discussion framework.

VERSION 1 – AUTHOR RESPONSE

Reviewer 1:	Krishnan, Vijay		
General 1.1	As a frequently ignored part of healthcare provision especially in more community-focussed societies, the participation of caregivers in healthcare is a topic that deserves attention. This is all the more important when the setting of the study involves children, and the risks are as severe as hospital-acquired infection in a population that is at significant risk.	We thank the reviewer for this comment, We have tried to emphasise this in mind doing the revision.	Introduction.
1.2	It is also reasonable to address this question through a qualitative approach (to elicit responses and observations that might not be found in a more structured or quantitative study) and to use multiple kinds of information-gathering (focussed group discussions, interviews, observation).	In the intro, we have added the following sentence: “Qualitative method was chosen as it is holistic and in-depth, exploring people's values and lived experiences and how these values are expressed (Tracy, 2024).”	Introduction 5th Paragraph.
1.3	To be useful to practitioners and researchers trying to develop a better understanding of the ways in which family caregivers may influence infection prevention and control, it would be needed to have new insights that are developed from the information	Thank you for your input. We understand your comment, we have now related the observations to the chosen theory and adjustments to highlight	Conclusion .<br><br>Limitations and strengths, 3rd Paragraph.<br><br>Discussion 6th Paragraph.

	gathered, and that can be generalised at least to other, similar settings. The authors should try not to focus too much on the specifics of the setting, and should avoid extensive summarisation of observations, so that they might identify common themes or ideas.	the relevance of the observations to the result and discussion.<br><br>Due to your comment, we have put more emphasis on the general implications of the results.	Results - Perceived Susceptibility and Severity 3rd and 4th Paragraph.
Introd. 1.4	Relevance of paragraph 2 of the introduction? (lines 40 - 47). May be relevant to the overall project, but does not link to the current paper	We have rephrased the paragraph to make the earlier research more relevant and increase the understanding for the reader.	Introduction 1st and 2nd Paragraph.
1.5	"Also poses a severe risk for the transmission of infectious pathogen" (["bmjpo-2025-004199_Proof_hi", p. 6](zotero://select/library/items/EQ5TQGCL)) ([pdf](zotero://open-pdf/library/items/GP5VYQ3K?page=6&annotation=4ZX69BYK)) \- would be useful to quantify this risk in terms of standard statistics (eg. attributable fractions)	This part was reformulated so that the focus now is on family caregivers' involvement, instead of the risk they pose.	Introduction 3rd Paragraph.
Methods 1.6	More information is needed on how the interviews were conducted, and what measures were taken to improve validity. Although some procedural factors (language, setting, stance) are mentioned, the authors report (FGDS: n= 5; 2-3 participants, 15-30 minutes; Interviews: n = 11; 9 - 20 minutes; lines 32 to 40, page 7) that the interactions were quite brief. It would be good to elaborate on specific steps that were used to elicit qualitative information and to confirm saturation (as this was the reported endpoint to terminate sample collection).	Thank you for your feedback, changes have been made to make this clearer.	Participants and Informants 3rd Paragraph.
1.7	It is unclear how the three information sources (FGDs, IIs, Obsevartions) interacted each other. It is also unclear whether the interview material was used in any way to identify specific areas for elaboration.	We thank the reviewer for this comment, but to keep focused, and with limited space, we have added a sentence to make this point.<br>In the methodology of this study, we used a thematically parallel study design using the HBM rather than being organized by data collection methods.	Methodology- Study design 1st Paragraph.
1.8	It appears that there were at least two interview teams. how was saturation assessed here? what	There was only one study team, which contained two participants. This has been revised to make that understandable for the reader.	Setting 3rd Paragraph.

1.9	Some material appears to be elicited from staff. The corresponding section in the methods (page 8; 10-16) does not provide much detail. for any interaction with staff. If staff were considered to be participants, please clarify this (how many interviews, were they also considered or FGDs or IIs? if not, what was the difference?	Thank you for your comment. Health care staff were informants, and not participants. Changes have been made to make that clearer.	Participants and Informants 7th Paragraph.
1.10	Please simplify and clarify the description of interviews in lines 37-44, page 7. the total number and distribution of interviews, along with participant characteristics, should be clearly described. at present, interview characteristics and participant characteristics are presented side-by-side.	Changes have been made to make this clearer, still in text form.	Participants and Informants 2nd and 3rd Paragraph.
1.11	What observations were made? very little anthropological observations/material are provided in the results or discussion. Mostly the observations presented are structural and institution-specific (layout, staffing, presence/absence of IPC material)	We have emphasised the use of social interactions, relationships between family caregivers towards each other and towards health care staff, mobile phones, kitchens and other factors contributing to transmission. If the reviewer would like more of similar observations please feel free to ask.	Please see Results
1.12	If a caregiver was excluded due to their not having sufficient contact, were any steps taken	We are not sure what the reviewer mean with “sufficient contact.” However, no caregiver was excluded beyond the exclusion criteria mentioned and not contact was deemed necessary with patients/caregivers excluded from the study	.
1.13	Essential details of the sample (proportion from neonatal/oncology setting, length of stay on the date of the interview) are not available.	Thank you for your feedback, this has been developed and clarified in the article.	Participants and Informants 2nd and 3rd Paragraph.
Results 1.14	Please separate FGD results, Interview results, observations here. As a qualitative study, it is important to differentiate sources and acknowledging subjectivity. This is missing in the presentation of results.	The results are presented thematically using the HBM rather than being organized by data collection methods. Due to your comment, this has been clarified.	Methodology- Study design 1st Paragraph.
Reviewer 2	Bouchoucha, Stephane L.		
General 2.1	Thank you for the opportunity to review this manuscript. A very important topic, under researched.	Thanks!	

2.2	The submission is well constructed and written. My only criticism would be that there are no obvious links made with the HBM in your discussion IT would be interesting to use the HBM as a discussion framework.	Thank you for your comment. Changes have been made in the discussion with an incorporated focus on the Health Belief Model.	Discussion.
